# Al-5Cu-0.3Sc-B_4_C Nanocomposites: Microstructural Refinement, Strengthening Mechanisms, and Corrosion Behavior

**DOI:** 10.3390/nano15231836

**Published:** 2025-12-04

**Authors:** Seyit Çağlar, Cengiz Temiz

**Affiliations:** 1 Department of Metallurgical and Materials Engineering, Zonguldak Bülent Ecevit University, Zonguldak 67100, Türkiye; 2 Department of Electronics and Automation, Alaplı Vocational School, Zonguldak Bülent Ecevit University, Zonguldak 67850, Türkiye; cengiztemiz@beun.edu.tr

**Keywords:** mechanical properties, precipitation strengthening, dispersion strengthening, nanocomposites, microgalvanic corrosion, wear mechanism

## Abstract

In this study, Al-5Cu-0.3Sc nanocomposites reinforced with 0–20 wt.% B_4_C were successfully fabricated using a combined melt-spinning, mechanical alloying, and sintering route. The rapid solidification achieved during melt spinning suppressed elemental segregation and refined the microstructure, producing a nanocrystalline Al-Cu-Sc matrix that served as a uniform host for B_4_C particles. X-ray diffraction confirmed the coexistence of Al, Al_2_Cu, Al_3_Sc, and B_4_C phases, indicating a dual-strengthening mechanism consisting of precipitation strengthening from Al_2_Cu/Al_3_Sc and particle strengthening from B_4_C. Increasing B_4_C content increased hardness from 44.9 HV to 188.2 HV (≈319%) via effective load transfer, interfacial dislocation accumulation, and particle–matrix interlocking. The wear rate decreased from 3.81 × 10^−3^ mm^3^/m to 6.29 × 10^−3^ mm^3^/m (≈98.35%), corresponding to a nearly 60-fold increase in wear resistance due to the formation of a stable ceramic tribofilm and the protective effect of embedded B_4_C particles. Conversely, the corrosion rate increased from 0.117 mm/year to 6.136 mm/year (≈52-fold) due to intensified microgalvanic interactions among B_4_C, Al_2_Cu, and the Al matrix. Generally, the incorporation of B_4_C reinforcement provides a great improvement in mechanical and tribological properties at the expense of corrosion resistance, highlighting a performance trade-off relevant for lightweight structural and surface critical applications.

## 1. Introduction

Aluminum-based alloys have become indispensable lightweight materials in modern engineering applications. The unique balance of low density, high strength-to-weight ratio, and superior formability makes these materials ideal for aerospace and automotive structures [1,2,3]. However, traditional Al and Al-Cu alloys have inherent limitations in high-temperature strength, thermal stability, and corrosion resistance, limiting their suitability for demanding operating environments. Consequently, great importance has been placed on the design of multi-component aluminum alloys that utilize microstructural refinement and precipitation engineering to enhance performance [4,5,6,7].

Among the alloying elements used in aluminum, scandium (Sc) is one of the most effective microalloying additions due to its strong grain-refining capability. Even small additions of Sc promote the formation of coherent Al_3_Sc precipitates with an L1_2_ crystal structure, which serve as efficient heterogeneous nucleation sites during rapid solidification. These nanoscale Al_3_Sc particles exert a pronounced stabilizing effect on dislocations and subgrain boundaries, thereby suppressing grain coarsening and improving both strength and thermal stability [8,9,10,11,12,13,14]. In addition, the presence of Al_3_Sc dispersoids contributes to the microstructural stability of the rapidly solidified Al-Cu matrix and helps maintain a refined structure during subsequent mechanical alloying and sintering. This microstructural stabilization indirectly complements the precipitation-hardening effect of Al_2_Cu, providing a combined strengthening response in the Al-Cu-Sc system [15,16,17].

Cu plays a vital role in strengthening aluminum alloys through precipitation hardening, primarily forming metastable θ′ (Al_2_Cu) and equilibrium θ (Al_2_Cu) phases during aging. The distribution, size, and consistency of these precipitates are greatly influenced by the Cu concentration and the heat treatment applied, which determine the overall mechanical performance of Al-Cu alloys. In Al-Cu-Sc systems, the interaction between Al_3_Sc and Cu-based precipitates alters the traditional precipitation sequence, yielding a more refined, thermally stable microstructure. This cooperative precipitation behavior increases strength without compromising ductility and provides superior thermal resistance compared to single-phase or binary Al-Cu alloys [18,19].

Boron (B) and carbon (C), typically added as boron carbide (B_4_C), further enhance the strengthening efficiency of aluminum-based alloys. One of the hardest known ceramic materials, B_4_C, combines high hardness with low density (2.52 g/cm^3^) and excellent thermal stability, making it an ideal reinforcement material for lightweight structural applications. When dispersed in an aluminum matrix, B_4_C contributes to improved hardness, elastic modulus, and wear resistance through effective load transfer, grain boundary constraint, and Orowan strengthening mechanisms [20,21,22,23]. Moreover, during high-energy grinding, B and C atoms can react with aluminum to form in situ Al_3_BC-type phases, which contribute to the refinement of the microstructure and further enhance the overall mechanical performance of the composite [24]. In this study, B_4_C particles with high purity and angular morphology were selected, as their shape promotes strong mechanical interlocking with the Al matrix. In contrast, their fine particle size enhances interfacial contact during consolidation. These characteristics make B_4_C highly effective for strengthening lightweight aluminum alloys without significantly increasing density.

Powder metallurgy (PM) and mechanical alloying, along with related powder-based techniques such as cold compaction and sintering, provide precise control over particle distribution, matrix-reinforcement interface bonding, and oxide film development. These parameters play a decisive role in determining the phase composition, solidification characteristics, and precipitation kinetics of nanostructured aluminum alloys, thereby managing their overall structural integrity and mechanical response [25,26,27]. Rapid solidification and high-energy grinding strongly influence the nucleation and growth behavior of Al_3_Sc phases, particularly in Sc-containing powders. These processing conditions alter the distribution and diffusion kinetics of the dissolved material, thereby affecting the overall microstructural development of the alloy and subsequently its aging response [28,29,30,31]. Although extensive studies have been conducted on Al-Sc and Al-Cu-Sc systems, the combined effect of B_4_C reinforcement on the microstructure and performance of Al-Cu-Sc nanocomposites has largely been unexplored. In particular, the interaction between ceramic reinforcements and precipitation behavior, as well as their collective effect on mechanical and corrosion properties, is not well understood. Furthermore, systematic investigations of the powder preparation parameters, sintering behavior, and phase stability of Al-5Cu-0.3Sc-B_4_C nanocomposites remain limited.

The melting-spinning technique was selected to achieve rapid solidification. This technique effectively suppresses element segregation, creating a nanocrystalline Al-Cu-Sc matrix with a homogeneous solute distribution. The refined microstructure provides an ideal basis for subsequent mechanical alloying and B_4_C reinforcement. The objective is to produce Al-5Cu-0.3Sc nanocomposites containing 5–20% by weight B_4_C and to systematically evaluate the effects of reinforcement content on microstructural evolution, hardness, wear behavior, and corrosion response.

Although B_4_C-reinforced aluminum composites have been widely examined across various alloy systems, the combined effects of rapid solidification, Sc microalloying, and ceramic reinforcement have not been systematically investigated in the Al-Cu-Sc-B_4_C quaternary system. Previous studies [25,32], including our earlier work on Al6061-B_4_C composites, have demonstrated that B_4_C can effectively enhance strength and wear performance; however, these systems do not incorporate the microstructural refinement achieved through melt spinning nor the unique precipitation/dispersoid effects associated with Sc additions. In contrast, the Al-Cu-Sc matrix used in this study undergoes ultrafast solidification, forming a nanocrystalline structure enriched with Al_3_Sc and Al_2_Cu strengthening phases. The interaction of this refined matrix with B_4_C particles has not been explored in the literature and represents a distinct scientific gap addressed in the present work.

This work introduces a novel hybrid reinforcement strategy that integrates rapid-solidification-induced Al_3_Sc dispersoids, precipitation strengthening from Al_2_Cu, and load-bearing ceramic reinforcement from B_4_C via a unified fabrication route. The resulting Al-5Cu-0.3Sc-B_4_C nanocomposites benefit from synergistic strengthening, achieving improved hardness, superior wear resistance, and stable structural integrity with minimal density increase. Owing to their combination of lightweight properties and enhanced performance, these materials hold strong potential for use in aerospace components, automotive engine systems, defense applications, and other sectors requiring durable, wear-resistant structural materials. The systematic evaluation presented here contributes to the broader understanding of next-generation aluminum-based nanocomposites. It clarifies the performance trade-offs inherent to incorporating ceramic reinforcement into Sc-containing Al alloys.

## 2. Materials and Methods

### 2.1. Preparation of Alloy and Ribbon Samples

The Al-5Cu-0.3Sc master alloy was first produced by induction melting under a controlled argon atmosphere to minimize oxidation. High-purity Al (99.9%) and Cu (99.9%) were charged into a ceramic crucible, along with a pre-alloyed Al-Cu-Sc bulk, and placed inside a sealed melt-spinning chamber equipped with a transparent protective cover to isolate the system from external contamination. The crucible was heated using an RF induction generator, enabling homogeneous melting through controlled, uniform energy input. Before ejection, the chamber was evacuated to 10^−4^ bar and subsequently back-filled with high-purity argon to establish an inert processing environment. The nozzle-wheel distance was adjusted to 0.20 mm to ensure stable melt flow. The alloy melt was then atomized onto a polished copper wheel rotating at 20 m/s, under an applied ejection pressure of 160 mbar. These parameters produced continuous ribbons with a typical width of ~10 mm, a thickness of ~20 µm, and a length of 3–10 cm. Due to the extremely high cooling rate (estimated at 10^5^–10^8^ K/s), solute redistribution was suppressed, and a refined nanocrystalline Al-Cu-Sc matrix was formed. The rapid solidification step also minimized segregation, producing chemically homogeneous ribbons suitable for subsequent powder processing and B_4_C integration.

### 2.2. Powder Preparation and Consolidation

The melt-spun ribbons were pulverized using a high-energy shaker mill (Chisun Tech, Guangzhou, China) to obtain fine, work-hardened powders. Milling was conducted for 10 h in an 80 mL hardened-steel vial using 15 mm diameter steel balls at a vibration frequency of 35 Hz. A 5:1 ball-to-powder weight ratio was selected based on previous optimization studies demonstrating that this ratio provides efficient impact energy transfer while avoiding excessive fracturing or cold-welding of aluminum powders. To prevent overheating and oxidation during milling, a 2 min rest followed a 5 min milling cycle, and the cycle was repeated continuously. The milled alloy powders were subsequently blended with B_4_C particles at 5–20 wt.% (Nanografi Nano Teknoloji AŞ, Ankara, Türkiye). According to the supplier’s technical specification sheet, the B_4_C reinforcement has a minimum purity of 95%, consisting of 77–79% B and 20–22% C, with impurity levels limited to ≤0.5% B_2_O_3_ and ≤0.2% Fe_2_O_3_. The powder has an average particle size of 16–24 μm, corresponding to a nominal 325-mesh classification, and exhibits an irregular angular morphology typical of high-hardness ceramic particulates. This combination of fine particle size, high purity, and angular shape enhances mechanical interlocking and promotes effective load transfer between the B_4_C particles and the aluminum matrix during consolidation. The blended powders were compacted under 600 MPa uniaxial pressure for 1 min to form 13 mm cylindrical green bodies. Sintering was performed in a Protherm vacuum furnace (Alser Teknik, Istanbul, Türkiye) under flowing argon at 625 °C for two hours, which is lower than the forming temperature of the new compounds [33,34]. This temperature was chosen because it is above the effective diffusion activation point of Al-Cu solid solutions yet below the threshold at which excessive grain coarsening or liquid-phase formation would occur. The selected dwell time promoted interparticle diffusion and bonding between the metallic and ceramic phases, resulting in mechanically stable compacts. Finally, the sintered pellets were ground with SiC abrasive papers (800–4000 grit) and polished using a 1 μm alumina suspension to obtain mirror-finished surfaces suitable for SEM, EDS, XRD, microhardness, wear, and electrochemical characterization.

It should be noted that the high-energy milling step in this study does not cause any chemical alloying between Al and B_4_C. Instead, the process aims to refine the rapidly solidified Al-Cu-Sc flakes, break up agglomerates, and achieve a uniform distribution of B_4_C particles through repeated fracture–cold welding cycles. No interfacial reaction products were detected by XRD, confirming that the milling process serves solely as a mechanical mixing step rather than a chemical alloying method.

### 2.3. Microstructural and Phase Characterization

Phase identification was performed using X-ray diffraction (XRD) analysis on a PANalytical Empyrean diffractometer (PANalytical, Almelo, The Netherlands) equipped with Cu-Kα radiation (λ = 1.5406 Å) and operating at 40 kV and 45 mA. Diffraction data were collected over 2θ from 10° to 90° with a step size of 0.02°. Surface morphology, particle distribution, and composition homogeneity were analyzed using an Oxford X-MaxN 80 energy-dispersive X-ray (EDX) detector (Oxford Instruments, Oxford, UK). The experimental densities of the nanocomposites were measured using a WSA-224 precision density kit (Bengbu Wuhua Instrument Co., Ltd., Bengbu, China) (based on Archimedes’ principle). Theoretical densities were calculated using the mixture rule based on the weight fractions of individual elements. Microstructural examinations of the B_4_C powders, sintered samples, and worn surfaces were performed using a JEOL S-6610 (JEOL Ltd., Tokyo, Japan) scanning electron microscope operating at an accelerating voltage of 15 kV. All samples were ultrasonically cleaned and coated with a thin Au-Pd conductive layer before imaging. The backscattered electron (BSE) mode was used to evaluate particle morphology, reinforcement distribution, and wear-track features. Elemental analysis and mapping were conducted using an attached EDX detector.

### 2.4. Mechanical Testing

Microhardness measurements were performed using a Shimadzu HMV-G21 Vickers hardness tester (Shimadzu Corporation, Kyoto, Japan) with an applied load of 0.98 N and a dwell time of 15 s. For each composition, ten indentations were made at randomly selected locations, and the mean value and standard deviation were calculated to ensure measurement reliability. Indentations that overlapped pores or edges were excluded. The indentation diagonals were kept within a consistent range of approximately 20–40 μm, which minimizes the indentation size effect (ISE). Since the same load was applied to all samples and the diagonal lengths remained within a stable range, no further ISE correction was required, and the reported values are directly comparable.

Dry sliding wear tests were performed using a TRIBOtechnic TRIBOtester (TRIBOtechnic, Montigny-le-Bretonneux, France) under a normal load of 11 N and a sliding distance of 100 m. All tests were conducted under ambient conditions at room temperature (25–33 °C) and a relative humidity of 29–36%. For each composition, three parallel wear tests were carried out under identical conditions, and the reported wear rate and wear resistance values correspond to the mean ± standard deviation of these repeated measurements. Wear scar profiles were recorded using a Taylor Hobson 2D profilometer (Taylor Hobson Ltd., Leicester, UK) to determine the cross-sectional area of the wear track. Wear volume and wear rate were calculated using the following equations:

(1) V=A×l

(2) $$WR=\frac{V}{S}$$

where *V* is the wear volume (mm^3^), *A* is the cross-sectional area of the wear track (mm^2^), *l* is the track length (mm), and *S* is the sliding distance (m). The wear rate (*WR*, mm^3^/m) was obtained from the slope of the wear volume–distance relationship.

### 2.5. Electrochemical Corrosion Tests

Electrochemical measurements were performed at 27 ± 2 °C in a 3.5 wt.% NaCl solution using a Gamry Reference 1010E potentiostat (Gamry Instruments, Warminster, PA, USA). A conventional three-electrode configuration was employed, comprising the nanocomposite sample as the working electrode, a saturated Ag/AgCl reference electrode, and a platinum counter electrode. Before each polarization test, the specimens were immersed in the NaCl solution until the open-circuit potential (OCP) stabilized, defined as a potential drift of <1 mV/min over 30 min, ensuring electrochemical equilibrium. The pH of the NaCl solution was checked before and after each test series and remained within 6.5–7.1, confirming solution stability throughout the measurements. The corrosion rate values reported in the Results section represent the mean ± standard deviation of these three repeated measurements. Potentiodynamic polarization tests were performed at a scan rate of 1 mV/s in the potential range of −0.5 V to +0.5 V. The corrosion current density (*I_corr_*) and corrosion potential (*E_corr_*) were determined by extrapolating the anodic and cathodic branches of the Tafel plots.

**Figure 1:**
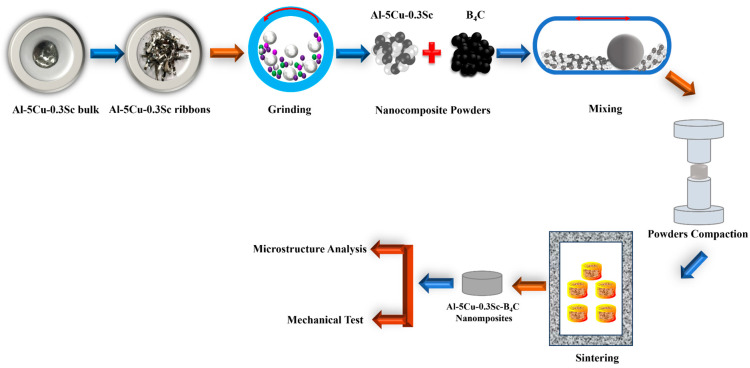
Schematic representation of the Al-Cu-Sc-B_4_C nanocomposite production and characterization.

The corrosion rate (*CR*) was calculated using Equation (3):

(3) $$CR=k\frac{I_{corr\;}EW}{\rho }$$

where *k* is a constant (3.27 × 10^−3^), *EW* is the equivalent weight (g.eq^−1^), ρ is the experimental density (g.cm^−3^), and *I_corr_* is the corrosion current density (µA.cm^−2^). A schematic representation of the overall experimental procedure, including alloy production, melt spinning, powder processing, sintering, and characterization, is shown in Figure 1.

## 3. Results and Discussion

**Figure 2:**
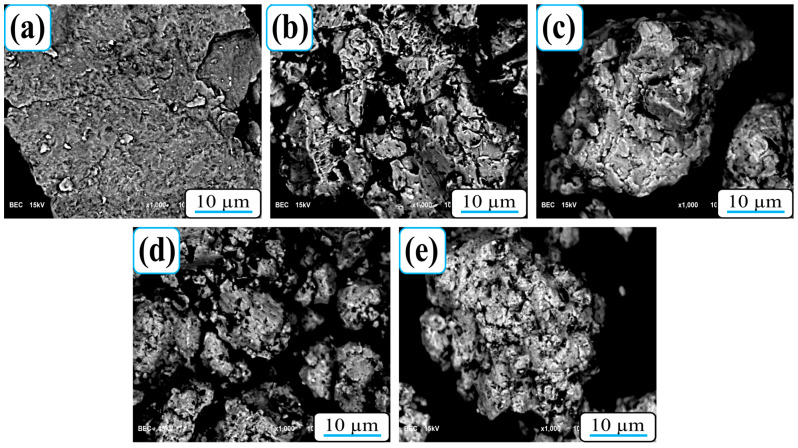
SEM micrographs of the composite powder mixtures before pressing for (**a**) 0B_4_C, (**b**) 5B_4_C, (**c**) 10B_4_C, (**d**) 15B_4_C, and (**e**) 20B_4_C nanocomposites.

The morphology of the composite powder mixtures before pressing was examined using SEM (Figure 2), confirming the uniform distribution of B4C within the Al-Cu-Sc matrix.

Table 1 shows the weight percentages of the Al-5Cu-0.3Sc-B_4_C nanocomposites synthesized in this study. The materials are denoted as 100-X(Al-5Cu-0.3Sc)-(X)B_4_C (X = 0, 5, 10, 15, and 20), where X = 0, 5, 10, 15, and 20 are weight percentages and are labeled as 0B_4_C, 5B_4_C, 10B_4_C, 15B_4_C, and 20B_4_C, respectively. The Al-5Cu-0.3Sc matrix obtained by the melt-spinning process exhibited an ultra-fine, nanocrystalline structure due to rapid solidification at a cooling rate of 10^5^ to 10^8^ K/s, while the added B_4_C reinforcement was in the micron size range. Therefore, the obtained materials can be defined as nanostructured aluminum-based composites reinforced with micro-sized ceramic B_4_C particles in a nanocrystalline metallic matrix. This hybrid structural configuration is expected to combine the high strength and stability of the nanocrystalline Al-Cu-Sc matrix with the internal hardness and wear resistance provided by B_4_C. The systematic variation in B_4_C content enables a comprehensive assessment of its effects on density, hardness, wear rate, and corrosion resistance, providing valuable insights for optimizing nanostructured aluminum composites for advanced engineering applications.

### 3.1. XRD Analysis

**Figure 3:**
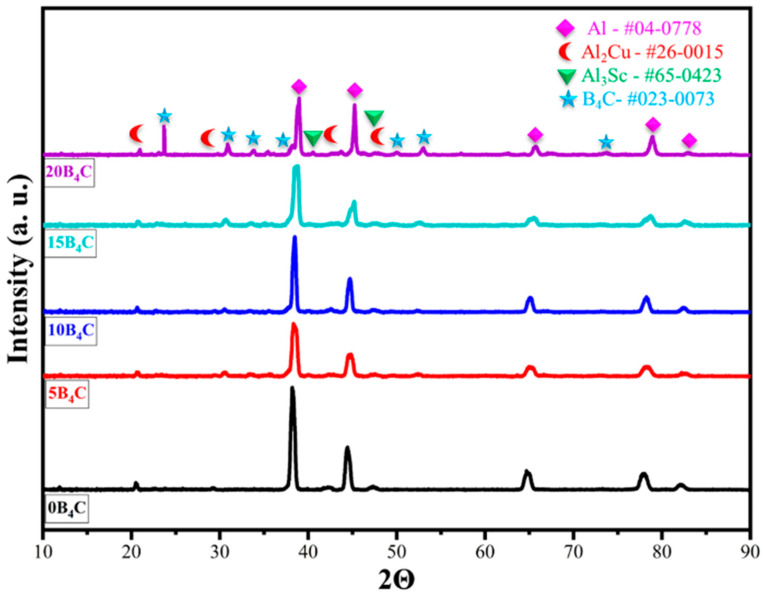
XRD pattern of Al-5Cu-0.3Sc-B_4_C nanocomposites.

Figure 3 shows the XRD patterns of Al-5Cu-0.3Sc-B_4_C nanocomposites with varying B_4_C contents (0–20 wt.%). The diffraction peaks observed at approximately 2θ = 38.4°, 44.7°, 65.1°, 78.2°, and 82.4 correspond to the (111), (200), (220), (311), and (222) planes of the face-centered cubic (FCC) Al phase (PDF#04-0778) (111), (200), (220), (311), and (222) planes and confirm that the aluminum matrix retains its crystalline structure after processing. In addition to the main Al reflections, weak peaks corresponding to the Al_2_Cu (PDF#26-0015) and Al_3_Sc (PDF#65-0423) phases were detected, indicating the precipitation of these secondary strengthening compounds within the matrix. The formation of Al_2_Cu indicates Cu segregation and precipitation during solid-state processing, while Al_3_Sc confirms the stability of Sc-rich precipitates that contribute to grain refinement and thermal stability [8]. With the addition of B_4_C, several low-intensity peaks at 2θ = 27.1°, 37.9°, and 41.6° were observed, corresponding to the B_4_C phase (PDF# 23-0073), confirming the successful incorporation of the ceramic reinforcement. No peaks associated with oxide or impurity phases were detected, indicating that the powder metallurgy and sintering processes were carried out under well-controlled atmospheric conditions. Furthermore, no diffraction peaks corresponding to Al_3_BC or any other Al-B_4_C reaction product were observed, confirming that the sintering temperature (625 °C) was insufficient to trigger interfacial reactions between Al and B_4_C. Thus, the B_4_C particles remained chemically stable and contributed primarily through mechanical reinforcement rather than interfacial phase formation.

As the B_4_C content increased, the intensity of the Al peaks decreased slightly, and the diffraction lines broadened moderately. This behavior indicates grain refinement and increased lattice stress resulting from the inclusion of hard ceramic particles and the mismatch in thermal expansion coefficients between the Al matrix and B_4_C. The presence of the Al_3_Sc phase is particularly important because, even at low concentrations, it contributes to grain boundary stabilization and enhances microstructural stability by forming consistent L1_2_-structured precipitates. Overall, the coexistence of Al, Al_2_Cu, Al_3_Sc, and B_4_C phases demonstrates a dual-strengthening mechanism that operates synergistically: precipitation hardening (via Al_2_Cu and Al_3_Sc) and dispersion hardening (via B_4_C particles). This phase configuration is consistent with microstructural observations and subsequent improvements in hardness and wear resistance.

The crystallite size (⟨D⟩) and lattice microstrain (⟨ε⟩) of the Al matrix were quantified using the Scherrer equation and peak-broadening analysis. For each composition, the dominant Al reflections ((111), (200), (220), (311), and (222)) were selected, and the corresponding 2θ positions and full width at half maximum (FWHM) values were used to calculate the crystallite size and strain. The Scherrer equation was used as follows [35]:

(4) $$D=\frac{K\lambda }{\beta \cos\theta }$$

where *K* = 0.94 is the shape factor, *λ* = 0.15406 nm (Cu Kα radiation), *β* is the measured FWHM (in radians), and *θ* is the Bragg angle.

The lattice microstrain (*ε*) was calculated from peak broadening using

(5) $$\varepsilon =\frac{\beta }{4\tan\theta }$$

The crystallite size and microstrain values obtained from individual peaks were averaged for each composition, and the resulting ⟨D⟩ and ⟨ε⟩ values are presented in Table 2. The results indicate that the Al matrix remains nanocrystalline across all compositions (⟨D⟩ ≈ 24–36 nm). Increasing B_4_C content generally decreases crystallite size, while the microstrain increases (from 2.11 × 10^−3^ to 3.34 × 10^−3^), confirming enhanced lattice distortion and defect accumulation at Al/B_4_C interfaces. It should also be noted that the slight anomaly in ⟨D⟩ at 15 wt.% B_4_C does not persist at 20 wt.%. This can be understood in terms of the competing effects of particle-induced fracturing and milling-induced heterogeneity. At 15 wt.% B_4_C, the reinforcement content is high enough to generate pronounced local stress fields and partial agglomeration, which promote heterogeneous deformation and may allow limited local recovery, leading to slightly coarser crystallites in some regions. At 20 wt.% B_4_C, however, the much higher fraction of hard particles intensifies repeated fracturing during mechanical alloying to such an extent that recovery is largely suppressed and a finer, more uniformly refined nanocrystalline matrix is obtained, even though the overall porosity remains relatively high. This competition between local heterogeneity and severe particle-assisted fragmentation provides a consistent explanation for the observed trend in ⟨D⟩.

### 3.2. Microstructural Characterization (SEM and EDX)

**Figure 4:**
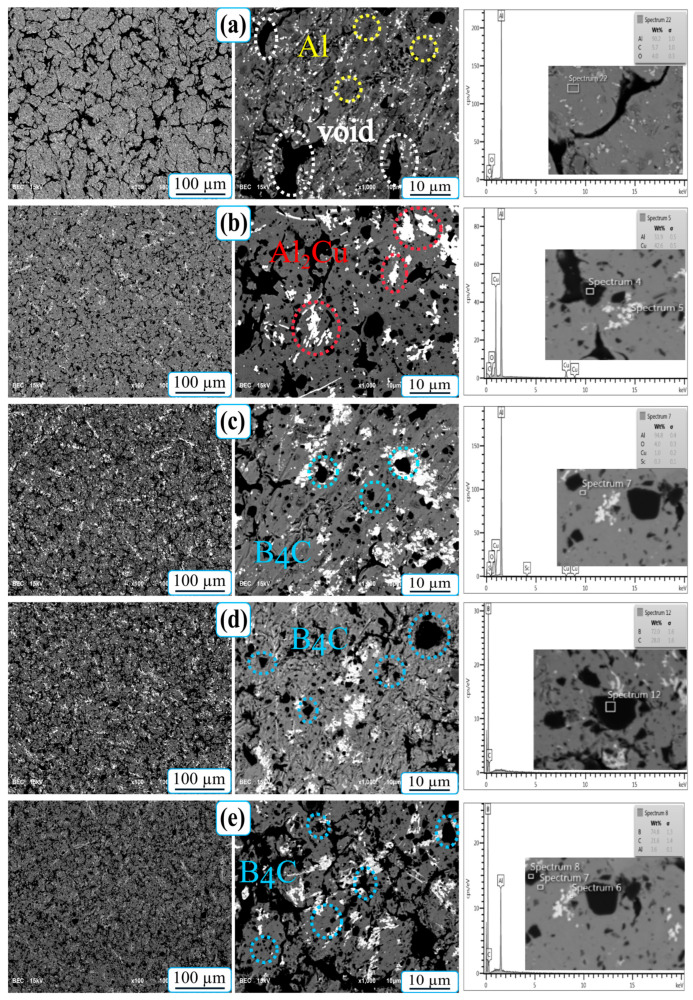
SEM-EDX micrographs of Al-5Cu-0.3Sc-B_4_C nanocomposites with different B_4_C contents, (**a**) 0B_4_C, (**b**) 5B_4_C, (**c**) 10B_4_C, (**d**) 15B_4_C, and (**e**) 20B_4_C, at magnifications of 100 µm and 10 µm.

**Figure 5:**
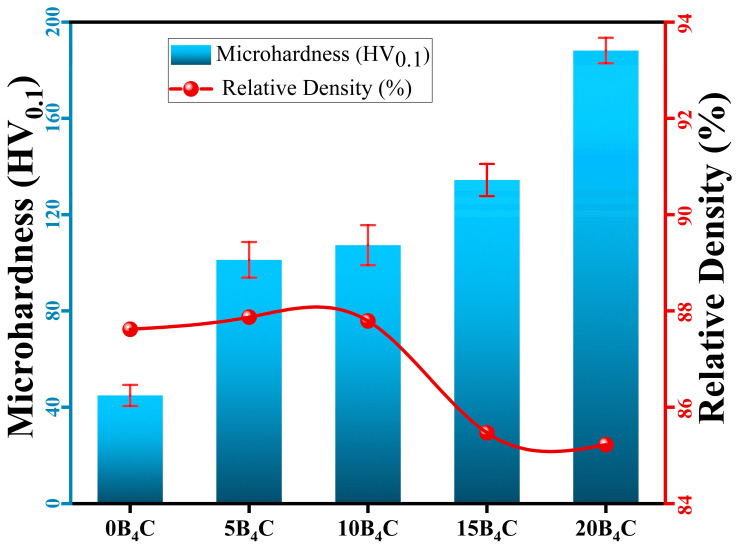
Variation in Vickers microhardness and relative density as a function of B_4_C content for Al-Cu-Sc-B_4_C nanocomposites.

SEM micrographs of Al-5Cu-0.3Sc-B_4_C nanocomposites at two magnification levels (100 µm and 50 µm), together with the corresponding EDS spot analyses, confirm the elemental composition of the identified phases (Al matrix, Al_2_Cu intermetallic, and B_4_C reinforcement). Although the nanoscale Al_3_Sc phase detected by XRD is not readily distinguishable in the SEM images due to its ultrafine dispersion, its presence is confirmed by the diffraction results shown in Figure 4 [8]. The unmodified sample (0B_4_C) exhibits a relatively disordered morphology with visible interparticle voids and loosely bonded regions between melted and curved particles. These voids indicate incomplete densification during sintering and limited plastic flow of the rapidly solidifying aluminum matrix. With the addition of B_4_C, the morphology becomes significantly more homogeneous, and the interparticle voids are markedly reduced. The dark, angular regions clearly observed in B_4_C-containing specimens correspond to well-dispersed ceramic reinforcement particles throughout the matrix and along grain boundaries. Their presence enhances interfacial bonding and limits grain coarsening, resulting in a denser, more stable microstructure. This observation is in good agreement with the improved relative density and hardness values reported in Table 3 and Figure 5.

In contrast, the brighter gray areas in the SEM images may represent Cu-rich intermetallic compounds (such as Al_2_Cu- or Al-Cu-Sc-type phases) formed during solidification and sintering. Given the low scandium content (0.3% by weight), Sc atoms are predominantly dissolved in the aluminum matrix. They are found as nano-scale Al_3_Sc precipitates rather than visible secondary phases at the given magnifications. Although these precipitates are not directly soluble, they contribute to grain refinement and microstructural stability. Overall, SEM observations confirm that B_4_C reinforcement effectively increases densification and microstructural homogeneity. Meanwhile, the rapid solidification induced by melt spinning helps preserve a fine-grained, defect-minimized matrix, providing an ideal foundation for the subsequent strengthening and corrosion performance of the composites.

### 3.3. Density and Microhardness Measurements

Figure 5 illustrates the relationship between relative density and Vickers microhardness for Al-5Cu-0.3Sc-B_4_C nanocomposites, with the corresponding results summarized in Table 3. Experimental measurements revealed that the relative density remained within a narrow range of 85.9–88.1%, whereas the microhardness increased continuously and significantly with increasing B_4_C content. Although the relative density values (85.9–88.1%) appear to fall within a narrow range, the incorporation of 5–20 wt.% B_4_C inherently promotes interfacial porosity due to the limited wettability between Al and the rigid ceramic particles. During pressing and sintering, the restricted flow of the Al matrix around angular B_4_C particles and the reduced interfacial diffusion capacity result in localized pore retention, particularly in B_4_C-rich regions. Minor gas entrapment originating from high-energy powder milling may further contribute to this effect. While these pores are small enough not to dominate the mechanical response, they provide a consistent explanation for the slight reduction in density at higher B_4_C contents and must also be considered when evaluating corrosion behavior, given the potential formation of microgalvanic sites [36,37]. The base alloy (0B_4_C) exhibited a microhardness of 44.9 HV, while this value gradually increased to 101.2 HV, 107.3 HV, 134.4 HV, and 188.2 HV for the 5B_4_C, 10B_4_C, 15B_4_C, and 20B_4_C nanocomposites, respectively. This corresponds to an overall hardness improvement of approximately 319% from 0 wt.% to 20 wt.% B_4_C.

Several complementary mechanisms govern the progressive increase in hardness with rising B_4_C content. First, B_4_C particles act as load-bearing reinforcements, restricting plastic deformation by transferring a portion of the applied stress to the ceramic phase. Their presence also induces strain incompatibility at the particle–matrix interfaces, increasing the density of geometrically necessary dislocations. Second, the rapid solidification process produces a refined Al matrix and promotes the formation of fine Al_2_Cu precipitates. The nanoscale Al_3_Sc dispersoids retained from melt spinning further stabilize this refined structure during mechanical milling and sintering, thereby limiting grain coarsening. The synergy of these mechanisms results in the observed steady strengthening trend.

At higher B_4_C levels, particularly at 20 wt.%, the sharp increase in hardness can be attributed to the dense distribution of hard ceramic particles, which significantly impede dislocation motion through the Orowan bypass mechanism in addition to particle–matrix load transfer. Although a slight reduction in relative density occurs at B_4_C contents above 10 wt.%, the reinforcement effects clearly outweigh the influence of porosity. The marginal density reduction is related to the limited wettability of B_4_C in aluminum and occasional particle agglomeration, which can locally restrict matrix flow during compaction and sintering.

The microhardness increased from 44.9 HV (0B_4_C) to 188.2 HV (20B_4_C), corresponding to an approximately 319% (4.19-fold) increase. This substantial enhancement reflects the combined contributions of load-bearing reinforcement, increased geometrically necessary dislocations, precipitation strengthening from Al_2_Cu, and the microstructural stabilization provided by nanoscale Al_3_Sc dispersoids. Although a slight reduction in density is observed at higher B_4_C contents, the strengthening imparted by the hard ceramic phase clearly outweighs the detrimental effects of porosity.

### 3.4. Wear Behavior

**Figure 6:**
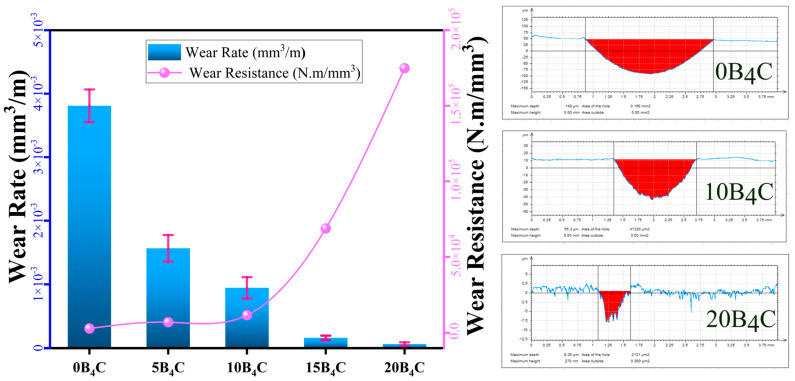
Variation in wear rate and wear resistance with B_4_C content for Al-Cu-Sc-B_4_C nanocomposites, together with representative profilometer cross-sectional wear-track profiles for 0B_4_C, 10B_4_C, and 20B_4_C samples.

Figure 6 shows the change in wear rate and wear resistance of the Al-5Cu-0.3Sc-B_4_C nanocomposites as a function of B_4_C content. As the amount of B_4_C increases, the profilometer cross-sectional wear tracks clearly become narrower and shallower, indicating a substantial reduction in material loss. The wear rate decreased steadily from 3.81 × 10^−3^ mm^3^/m for the unmodified alloy (0B_4_C) to 6.29 × 10^−5^ mm^3^/m for the nanocomposite containing 20% B_4_C by weight. Conversely, wear resistance increased sharply from 2.89 × 10^−3^ mm^3^/m to 1.75 × 10^−5^ mm^3^/m over the same composition range. This strong inverse relationship clearly demonstrates the dominant strengthening and tribological improvement provided by B_4_C reinforcement. The marked decrease in wear rate with increasing reinforcement content can be attributed to the hard and chemically stable structure of B_4_C particles, which act as load-bearing components during sliding contact. These particles minimize the direct interaction area between the counter surface and the Al matrix, suppressing adhesive wear and plastic deformation. In parallel, the increased microhardness of the nanocomposites (Figure 5) enhances resistance to friction cutting and surface material loss. The sharp drop in wear rate above 10% by weight of B_4_C indicates a transition dominated by particle–matrix load transfer and interfacial bonding, forming a robust barrier against material loss.

At low B_4_C content (≤5% by weight), the wear mechanism is primarily adhesive and abrasive, driven by partial exposure of the soft Al-Cu matrix and limited reinforcement coverage of the wear surface. At higher reinforcement levels (10–15 wt.%), the wear mechanism transitions to a mixed mode characterized by micro-shearing and shallow abrasive grooves. The 20B_4_C nanocomposite exhibits the lowest wear rate and highest wear resistance, indicating a predominantly light abrasive mechanism with limited plastic flow. The improvement in tribological performance at this stage stems from the combined effects of dispersion strengthening, grain refinement, and the formation of a mechanically stable tribo-layer. The results generally confirm that increasing the B_4_C content significantly enhances the wear resistance of Al-5Cu-0.3Sc nanocomposites. The 15–20% B_4_C weight range provides an optimal balance between hardness and compactness, effectively minimizing material loss during sliding. These findings are consistent with the hardness trend and confirm that wear resistance is primarily determined by the degree of dispersion strengthening and the microstructural stability of the composite surface.

As a result, the progressive decrease in wear rate from 3.81 × 10^−3^ mm^3^/m to 6.29 × 10^−5^ mm^3^/m and the corresponding ~60-fold increase in wear resistance demonstrate that B_4_C reinforcement significantly improves the tribological behavior of the Al-5Cu-0.3Sc matrix. This improvement is primarily governed by the load-bearing effect of B_4_C particles, reduced plastic deformation, and the transition from severe adhesive wear to predominantly abrasive and mild oxidative wear as the reinforcement content increases.

**Figure 7:**
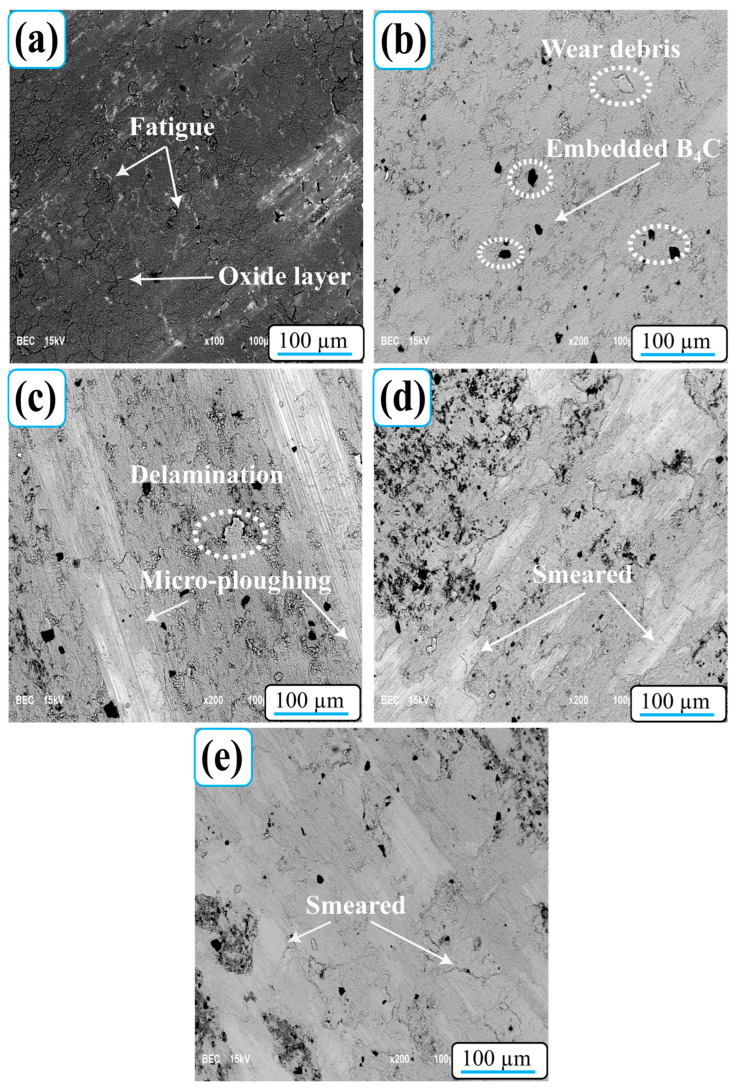
SEM images of the worn surfaces of (**a**) 0B_4_C, (**b**) 5B_4_C, (**c**) 10B_4_C, (**d**) 15B_4_C, and (**e**) 20B_4_C Al-5Cu-0.3Sc-B_4_C nanocomposites.

Figure 7 shows SEM micrographs of the worn surfaces of Al-5Cu-0.3Sc-B_4_C nanocomposites tested at 10 N, revealing that the wear mechanisms evolved progressively with increasing B_4_C content. The surfaces exhibit distinct morphological features, including adhered layers, delamination regions, oxide films, and embedded reinforcement particles, each of which correlates with the mechanical and tribological properties shown in Figure 7. The unreinforced alloy (0B_4_C, Figure 7a) exhibits pronounced plastic deformation and extensive contamination layers, typical features of surface adhesive wear. As a result of repeated adhesion–fracture cycles between the soft Al-Cu matrix and the steel counterball, large delamination areas and accumulated wear debris are evident. During sliding, local temperature increases promote oxidative wear, leading to the formation of continuously fracturing and reforming thin Al_2_O_3_-based tribofilms. The combined effects of plastic flow and oxide fatigue result in a rough morphology characterized by adhesive oxidative delamination.

At a 5% B_4_C weight ratio (Figure 7b), delamination becomes more localized, and shallow grooves begin to appear, indicating a transition to a mixed adhesive–abrasive wear mode. Dispersed B_4_C particles function as load-carrying regions, reducing plastic deformation and limiting severe adhesion. However, partial particle agglomeration and subsurface stress concentration can still initiate fatigue cracks, which propagate to the surface, forming small delamination flakes and oxidized residue clusters. At higher reinforcement levels (10–20% B_4_C by weight; Figure 7c–e), the worn surfaces become significantly smoother, with fewer delamination regions. On the surface, fine micro-scratch grooves dominate, along with isolated embedded B_4_C particles and a thin mechanically mixed layer (MML). This layer forms through the mechanical entrapment of oxidized residues and hard particles, acting as a self-lubricating tribofilm that reduces direct metal-to-metal contact. As a result, the primary wear mechanism shifts toward abrasive wear, becoming consistent with significant improvements in hardness and wear resistance (Figure 5). Overall, the wear mechanism evolves from adhesive–oxidative delamination in the unmodified alloy to abrasive wear in highly reinforced nanocomposites. The inclusion of B_4_C not only increases hardness and load-carrying capacity but also stabilizes the oxide layer, reducing fatigue-induced delamination.

**Figure 8:**
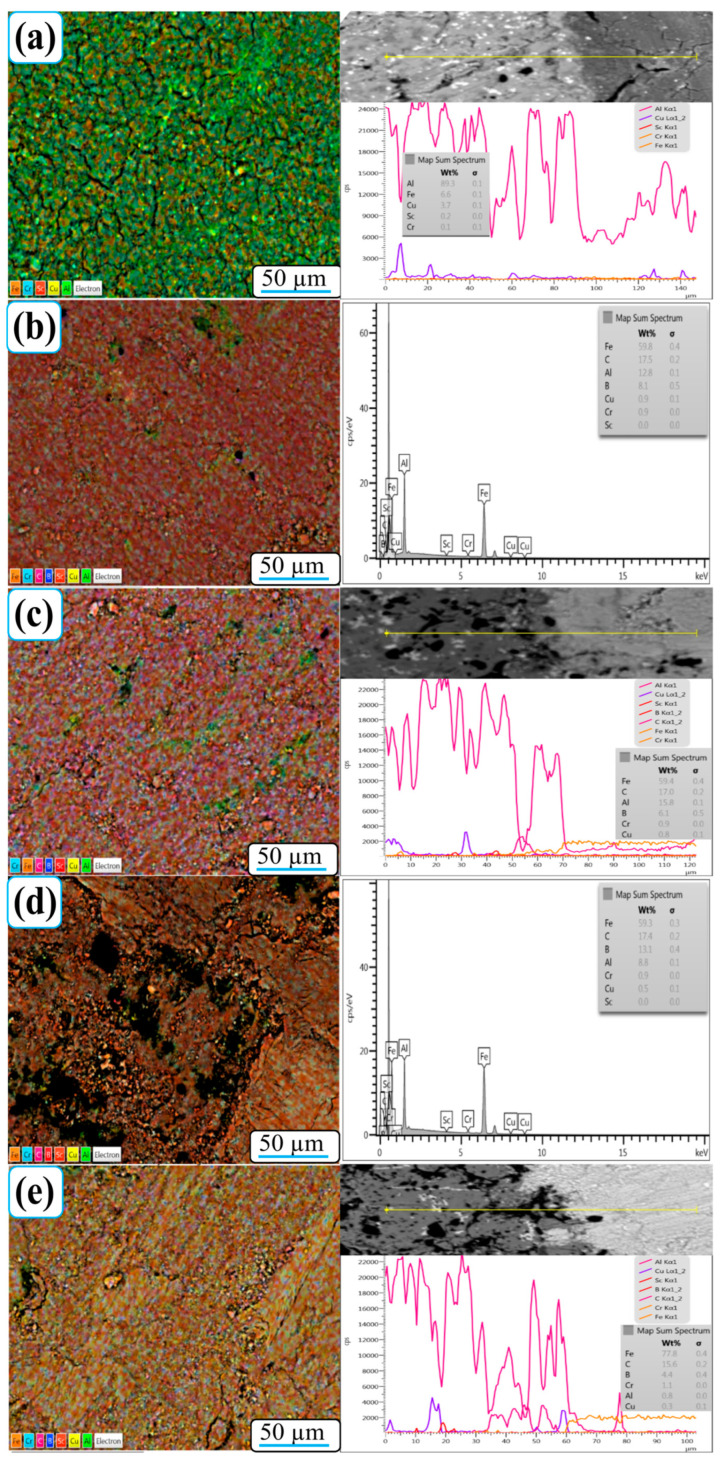
SEM-EDX elemental mapping images of the worn surface of (**a**) 0B_4_C, (**b**) 5B_4_C, (**c**) 10B_4_C, (**d**) 15B_4_C, and (**e**) 20B_4_C Al-5Cu-0.3Sc-B_4_C nanocomposites. EDS line-scan profiles in (**a**), (**c**), and (**e**) trace the transition from unworn to worn regions, showing increased Fe/Cr transfer and B_4_C-related features within the wear track.

Figure 8 shows SEM-EDX element mapping images of the worn surfaces of Al-5Cu-0.3Sc-B_4_C nanocomposites with varying B_4_C contents (0–20 wt.%). The element maps reveal a homogeneous distribution of Al, Cu, Sc, B, and C across all samples, confirming that the processing route used effectively promoted the uniform dispersion of reinforcement particles within the aluminum matrix. Minor fluctuations observed in the B and C signals are attributed to EDX’s inherent limitations in detecting low-atomic-number (light) elements, rather than to actual compositional inhomogeneity [38]. This ensures that the detected distribution trends reliably represent the true spatial distribution of B_4_C within the nanocomposites.

For the 0B_4_C nanocomposite (Figure 8a), the worn surface predominantly contains Al, Cu, and O, indicating the formation of a thin oxide film during sliding. The local enrichment of Fe and Cr is minimal, indicating limited material transfer from the steel counterball. As the B_4_C content increases (Figure 8b–e), the surface distribution of Fe, Cr, and C becomes more pronounced. This trend implies intensified friction interaction with the counter surface, leading to microfractures in the steel ball and subsequent transfer of Fe- and Cr-rich residues to the composite surface. These transferred elements tend to accumulate around B_4_C-rich regions, facilitating oxidation and mechanical embedding within the localized heating wear trace.

The presence of Fe- and Cr-enriched oxide islands, along with oxygen-rich regions, supports the formation of a protective tribo-oxide layer that minimizes metal-to-metal contact and reduces adhesive wear. Particularly in 15B_4_C and 20B_4_C nanocomposites, higher reinforcement content promotes the development of a more stable mechanical mixing layer (MML) composed of Al, B, C, Fe, and O. This compact oxide–ceramic film acts as a self-lubricating barrier, consistent with the significant reduction in wear rate and increased wear resistance reported in Figure 6. Therefore, EDX mapping results clearly show that the inclusion of B_4_C not only increases element homogeneity within the nanocomposite but also promotes the formation of a composite tribofilm consisting of oxide, metallic, and ceramic phases. This tribo layer plays a dominant role in wear protection by stabilizing surface reactions, evenly distributing stress, and preventing severe delamination during sliding.

In addition to the area maps, EDS line-scan profiles were obtained for the 0B_4_C, 10B_4_C, and 20B_4_C specimens, traversing from the unworn surface into the wear track. These profiles clearly demonstrate the compositional transition caused by sliding. The unworn region exhibits strong Al and Cu signals, whereas the worn area shows significant enrichment of Fe and Cr due to counterbody transfer. B and C intensities also increase locally within the wear track as fragmented B_4_C particles become exposed and incorporated into the mechanically mixed layer. This direct comparison between unworn and worn regions confirms the tribo-chemical evolution captured in the mapping results.

### 3.5. Corrosion Behavior

**Figure 9:**
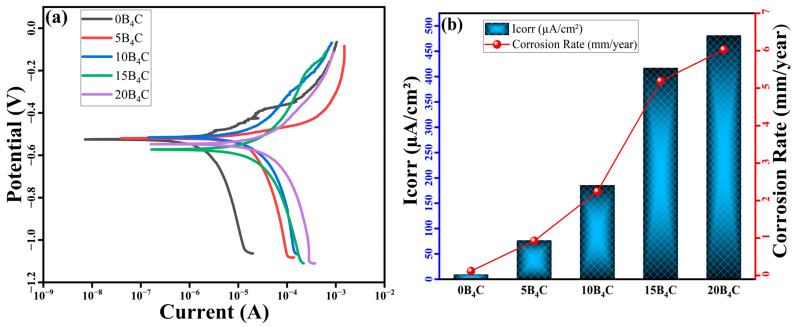
(**a**) Potentiodynamic polarization curves, (**b**) Variation in corrosion current density *I_corr_* and corrosion rate with B_4_C content for Al-Cu-Sc-B_4_C nanocomposites.

Figure 9a shows the potentiodynamic polarization curves of Al-5Cu-0.3Sc-B_4_C nanocomposites, while the relevant electrochemical parameters are summarized in Figure 9b and Table 4. The corrosion potential (*E_corr_*) and corrosion current density (*I_corr_*) clearly demonstrate the effect of B_4_C content on the corrosion behavior of the nanocomposites. The base alloy (0B_4_C) exhibited the most noble corrosion potential (−525 mV) and the lowest current density (9.68 µA cm^−2^), corresponding to a corrosion rate of 0.117 mm/year. However, with increasing B_4_C content, both *I_corr_* and the corrosion rate gradually increased, reaching 481.6 µA cm^−2^ and 6.14 mm/year, respectively, for the 20B_4_C nanocomposite.

This trend suggests that while adding B_4_C enhances hardness and wear resistance, it reduces corrosion performance. The presence of B_4_C creates additional interfaces between the ceramic reinforcement and the metallic matrix. These interfaces, which have different electrochemical potentials, function as microgalvanic cells when exposed to chloride-containing electrolytes, thereby accelerating the anodic dissolution of the aluminum matrix. Increased porosity at higher B_4_C loadings (Table 3) further facilitates electrolyte ingress, promoting localized corrosion along particle–matrix boundaries.

Polarization curves reveal that all nanocomposites exhibit active corrosion behavior, characterized by the absence of a significant passivation region, unlike aluminum alloys in chloride environments. The similar slopes of the cathodic branches in all samples indicate that oxygen reduction remains the dominant cathodic process. In contrast, the anodic response is strongly influenced by microgalvanic coupling associated with B_4_C interfaces [39,40,41,42]. The shift of *E_corr_* toward more negative values and the significant increase in *I_corr_* at a B_4_C weight ratio above 10% confirm that particle agglomeration and interfacial defects formed during sintering disrupt the continuity of the protective oxide film. Despite the observed decrease in corrosion resistance, nanocomposites maintain acceptable electrochemical stability for structural applications where mechanical and wear performance are prioritized. The overall results emphasize a balance between tribological improvement and corrosion resistance, highlighting the importance of optimizing B_4_C distribution during processing and minimizing interfacial porosity to achieve balanced performance [32,43].

As a result, the corrosion rate increased from 0.117 mm/year (0B_4_C) to 6.136 mm/year (20B_4_C), corresponding to an approximately 52-fold increase. This strong deterioration in corrosion resistance is attributed to microgalvanic coupling between B_4_C particles, the Al matrix, and Al_2_Cu intermetallics. The presence of ceramic particles locally disrupts the passive film and increases galvanic interfaces, while the Al_2_Cu phase acts as a cathodic site, accelerating localized dissolution. The combined effect of passive film discontinuity and galvanic interactions results in progressively higher corrosion rates as B_4_C content increases.

In addition to microgalvanic coupling, the corrosion response is strongly influenced by the intrinsic porosity of the PM-processed composites. The 85.9–88.1% relative density promotes electrolyte penetration and local discontinuities in the oxide film, which accelerate pit initiation. Although B_4_C is only weakly conductive, literature indicates that it behaves as a slightly cathodic phase relative to Al, enhancing matrix dissolution at the particle–matrix interface. This mild cathodic shift is sufficient to drive localized anodic dissolution of the surrounding Al when exposed to chloride electrolytes, causing B_4_C-rich regions to act as microgalvanic initiation sites. The effect becomes more pronounced when porosity or interfacial defects facilitate electrolyte access to the Al/B_4_C interface. The absence of Al_3_BC or related phases in XRD confirms that the chosen sintering temperature avoided interfacial reaction products that could further alter electrochemical behavior. Therefore, the overall corrosion tendency arises from the synergistic effects of porosity-driven oxide disruption and mild B_4_C-induced microgalvanic coupling.

The deeper analysis of hardness, wear behavior, and corrosion response confirms that the combined effects of reinforcement-induced microstructural refinement, load-bearing strengthening, and microgalvanic interactions govern the performance evolution of the Al-Cu-Sc-B_4_C system.

### 3.6. Synergistic Strengthening Mechanism of Sc and B_4_C

The mechanical performance of the Al-Cu-Sc-B_4_C nanocomposites arises from the cooperative interaction between Al_3_Sc nanoprecipitates and B_4_C ceramic particles, which contribute through distinct yet complementary strengthening pathways. During rapid solidification, the formation of fine and thermally stable Al_3_Sc dispersoids provides effective Zener pinning, suppressing grain boundary migration and preserving a refined matrix capable of sustaining high dislocation densities. This refined structure enhances the alloy’s intrinsic ability to accommodate subsequent reinforcement introduced during mechanical milling. Simultaneously, B_4_C particles generate strong strain gradients at the particle–matrix interfaces, leading to the formation of a high density of geometrically necessary dislocations. These dislocations not only impede plastic flow directly through Orowan looping but also promote the nucleation of secondary Al_2_Cu and Al_3_Sc precipitates, further intensifying precipitation-based strengthening. At higher B_4_C fractions, the interaction becomes increasingly synergistic: the matrix stability ensured by Al_3_Sc enables effective load transfer, while B_4_C-induced dislocation accumulation enhances the precipitation response. This dual mechanism explains both the substantial hardness increment (from 44.9 HV to 188.2 HV; +319%) and the >98% reduction in wear rate observed in the developed composites. Collectively, the integration of thermally stable Al_3_Sc dispersoids with rigid B_4_C reinforcements establishes a multiscale strengthening network—grain refinement, precipitation strengthening, load transfer, and dislocation hardening—which governs the superior mechanical and tribological behavior of the alloy system.

Beyond mechanical enhancement, this synergy enables the material to meet the functional requirements of advanced lightweight engineering applications. The achieved hardness, high wear resistance, stable tribofilm formation, and improved surface durability make these nanocomposites suitable for components subjected to repeated frictional loading, such as automotive piston skirts, cylinder liners, sliding bearings, and other tribologically loaded machine elements. Furthermore, the combined thermal stability of Al_3_Sc and the high softening resistance of B_4_C indicate potential for service in moderately elevated-temperature environments. While additional optimization, such as tailored heat treatment or B_4_C surface modification, could further expand the performance window, the present results demonstrate that the Al-Cu-Sc-B_4_C system offers a competitive balance of low density, high strength, and wear resistance suitable for next-generation structural and tribological applications.

## 4. Conclusions

Al-5Cu-0.3Sc-B_4_C nanocomposites were successfully produced through melt-spinning, followed by mechanical milling and sintering, which enabled a uniform microscale distribution of B_4_C particles within the aluminum matrix. XRD analysis confirmed the presence of Al, Al_2_Cu, Al_3_Sc, and B_4_C phases, indicating that both precipitation strengthening (Al_3_Sc and Al_2_Cu) and dispersion strengthening contributed to the overall mechanical response.

The hardness increased from 44.9 HV (0B_4_C) to 188 HV (20B_4_C), reflecting the combined influence of load-bearing particle reinforcement, dislocation accumulation, and the presence of strengthening precipitates. Wear tests showed a significant improvement in wear resistance with increasing B_4_C content, and SEM-EDS analyses demonstrated the formation of a mixed tribo-oxide layer containing Al-O species and Fe/Cr-rich transfer debris from the steel counterbody. This mechanically mixed layer is consistent with the reduced wear-track depth and the transition toward a more stable abrasive–oxidative wear regime at higher reinforcement levels.

Electrochemical measurements revealed a moderate increase in Icorr and corrosion rate with increasing B_4_C, attributed to microgalvanic interactions between the ceramic particles and the Al matrix, along with the higher porosity observed at elevated reinforcement ratios.

Summaries, the synergistic effects of Sc addition and B_4_C reinforcement enhanced strengthening, promoted stable tribofilm formation during sliding, and delivered simultaneous improvements in mechanical and tribological performance. These characteristics highlight the potential of Al-Cu-Sc-B_4_C nanocomposites for lightweight, wear-resistant applications that require a balance of strength, stability, and surface durability.

Limitations of the Study: This study is limited by the use of microscopy techniques with insufficient resolution to directly resolve nanoscale features such as grain boundaries, Al_3_Sc nanoprecipitates, and the internal structure of the mechanically mixed layer. TEM, EBSD, and high-resolution STEM-EDX analyses would be necessary to validate the proposed strengthening and wear mechanisms fully. Additionally, the influence of reinforcement-induced porosity on corrosion behavior requires further investigation using electrochemical impedance spectroscopy (EIS) and post-corrosion surface characterization. These limitations will be addressed in future work.

## Figures and Tables

**Table 1:** Chemical compositions of the Al-Cu-Sc-B_4_C nanocomposites.

	Chemical Composition (wt.%)			
	Al	Cu	Sc	B_4_C
**Al-5Cu-0.3Sc**	94.7	5	0.3	0
**Al-5Cu-0.3Sc-5B_4_C**	89.715	5	0.285	5
**Al-5Cu-0.3Sc-10B_4_C**	84.730	5	0.27	10
**Al-5Cu-0.3Sc-15B_4_C**	79.745	5	0.255	15
**Al-5Cu-0.3Sc-20B_4_C**	74.760	5	0.24	20

**Table 2:** Average crystallite size (⟨D⟩) and microstrain (⟨ε⟩) of the Al matrix in the Al-Cu-Sc-B_4_C nanocomposites.

	Crystallite Size ⟨D⟩ (nm)	Microstrain ⟨ε⟩ (×10^−3^)
**0** **B_4_C**	36.4	2.11
**5** **B_4_C**	30.3	2.42
**10** **B_4_C**	27.7	2.61
**15** **B_4_C**	29.5	2.52
**20** **B_4_C**	24.5	3.34

**Table 3:** Theoretical and experimental density values (mean ± standard deviation), relative density, and porosity of the Al-Cu-Sc-B_4_C nanocomposites.

	Theoretical Density (g/cm^3^)	Experimental Density (g/cm^3^)	Std. Dev. (g/cm^3^)	Relative Density (%)	Porosity (%)
**0** **B_4_C**	2.798	2.452	0.026	87.65	12.35
**5** **B_4_C**	2.781	2.45	0.032	88.09	11.91
**10** **B_4_C**	2.767	2.439	0.033	88.14	11.86
**15** **B_4_C**	2.752	2.366	0.034	85.97	14.03
**20** **B_4_C**	2.737	2.350	0.039	85.88	14.12

**Table 4:** Polarization results of the Al-Cu-Sc-B_4_C nanocomposites.

Materials	*E_corr_* (mV)	*I_corr_* (µA/cm^2^)	Corrosion Rate (mm/year)
**0** **B_4_C**	−525	9.68	0.117
**5** **B_4_C**	−515	185.78	2.263
**10** **B_4_C**	−573	415.78	5.095
**15** **B_4_C**	−520	426.31	5.391
**20** **B_4_C**	−547	481.57	6.136

## Data Availability

The original contributions presented in this study are included in the article. Further inquiries can be directed to the corresponding authors.

## References

[1] Tian L.P., Quan L.W., Wang C.C., Yu W., Huang L.X., Shin K.S., Yu H. (2023). Microstructure and Mechanical Properties of 2524 Aluminum Alloy with Dislocation Loops by Various Quenching Rates. Mater. Sci. Eng. A.

[2] Murty B.S., Kori S.A., Chakraborty M. (2002). Grain Refinement of Aluminium and Its Alloys by Heterogeneous Nucleation and Alloying. Int. Mater. Rev..

[3] Sun T., Luo Z., Duan Y., Zhang J. (2025). Preparation and Properties of High-Toughness AlMgB_14_ Material. Nanomaterials.

[4] Li Y., Liu Z., Xia Q., Liu Y. (2007). Grain Refinement of the Al-Cu-Mg-Ag Alloy with Er and Sc Additions. Metall. Mater. Trans. A.

[5] El-Mahallawy N.A., Taha M.A. (1987). Melt Spinning of Al-Cu Alloys: Modelling of Heat Transfer. J. Mater. Sci. Lett..

[6] Hao X., Jiaming L., Zhiqi W., Junyuan B., Zhihao Z., Gaowu Q. (2024). Achieving High Strength of Al-Cu-Li-Sc Alloy over Wide Temperature Range from 25 °C to 300 °C. Mater. Sci. Eng. A.

[7] Wang X., Cheng G., Zhang Y., Wang Y., Liao W., Venkatesh T.A. (2022). On the Evolution of Nano-Structures at the Al–Cu Interface and the Influence of Annealing Temperature on the Interfacial Strength. Nanomaterials.

[8] Temiz C., Yılmaz F., Çağlar S., Kölemen U., Uzun O. (2025). Influence of Sc Addition and Rapid Solidification on the Microstructure and Mechanical Properties of Al-5Cu Alloys. Phys. Scr..

[9] Beresina A., Chuistov K., Monastyrskaya T., Schmidt U. (2002). Structure of Rapidly Quenched Al-Sc Alloys. Mater. Technol..

[10] Torma T., Kovács-Csetényi E., Turmezey T., Ungár T., Kovács I. (1989). Hardening Mechanisms in Al-Sc Alloys. J. Mater. Sci..

[11] Wang Q. (2024). A Direct Particle Tracking Model for Predicting Homogeneous Precipitation Kinetics in Al-Sc Alloys. Comput. Mater. Sci..

[12] Ahmad Z. (2003). The Properties and Application of Scandium-Reinforced Aluminum. JOM.

[13] Dorin T., Ramajayam M., Vahid A., Langan T. (2018). Aluminium Scandium Alloys. Fundamentals of Aluminium Metallurgy: Recent Advances.

[14] Huang S., Luo S., Qin L., Shu D., Sun B., Lunt A.J.G., Korsunsky A.M., Mi J. (2022). 3D Local Atomic Structure Evolution in a Solidifying Al-0.4Sc Dilute Alloy Melt Revealed in Operando by Synchrotron X-Ray Total Scattering and Modelling. Scr. Mater..

[15] Dorin T., Ramajayam M., Lamb J., Langan T. (2017). Effect of Sc and Zr Additions on the Microstructure/Strength of Al-Cu Binary Alloys. Mater. Sci. Eng. A.

[16] Zhao J.-R., Lee L.-Y., Chang K.-C., Hung F.-Y. (2022). A Novel Two-Stage Heat Treatment with Medium-Temperature Aging Influence on Microstructure, Al_3_(Sc, Zr) Nanoprecipitation, and Application Properties, Enhancing Selective Laser Melting of Al–Mg–Sc–Zr Alloy. Nanomaterials.

[17] Wang Y., Li Y., Zhang T., Wang H., Zou H., Gao B., Tang H. (2025). Effects of Scandium on the Microstructure and Mechanical Properties of 2524 Aluminum Alloy. J. Alloys Compd..

[18] Yan J., Xiong X.K., Wu C.L., Ming W.Q., Xie P., Chen J.H. (2025). A Secondary High-Temperature Precursor of the Θ′-Phase in Al-Cu-(Sc) Alloys. J. Mater. Sci. Technol..

[19] Qin J., Tan P., Quan X., Liu Z., Yi D., Wang B. (2022). The Effect of Sc Addition on Microstructure and Mechanical Properties of As-Cast Zr-Containing Al-Cu Alloys. J. Alloys Compd..

[20] Guo H., Zhang Z., Zhang Y., Cui Y., Sun L., Chen D. (2020). Improving the Mechanical Properties of B_4_C/Al Composites by Solid-State Interfacial Reaction. J. Alloys Compd..

[21] Chand S., Chandrasekhar P., Roy S., Singh S. (2021). Influence of Dispersoid Content on Compressibility, Sinterability and Mechanical Behaviour of B_4_C/BN Reinforced Al6061 Metal Matrix Hybrid Composites Fabricated via Mechanical Alloying. Met. Mater. Int..

[22] Lin Q., Shen P., Qiu F., Zhang D., Jiang Q. (2009). Wetting of Polycrystalline B_4_C by Molten Al at 1173–1473K. Scr. Mater.

[23] Çağlar S. (2025). Enhancing Structural, Mechanical, and Radiation-Shielding Properties of Al-B_4_C Hybrid Composites. Sustainability.

[24] Maity T., Prakash A., Roy D., Prashanth K.G. (2025). In Situ Al_3_BC/Al Composite Fabricated via Solid-Solid Reaction: An Investigation on Microstructure and Mechanical Behavior. Appl. Sci..

[25] Çağlar S., Gaylan Y. (2025). Optimization of the Microstructural, Mechanical, and Radiation Shielding Properties of Al-30B_4_C-25 W Hybrid Composites with Gd_2_O_3_ Reinforcement. Sci. Rep..

[26] Gogebakan M., Kursun C., Eckert J. (2013). Formation of New Cu-Based Nanocrystalline Powders by Mechanical Alloying Technique. Powder Technol..

[27] Khakbiz M., Akhlaghi F. (2009). Synthesis and Structural Characterization of Al–B_4_C Nano-Composite Powders by Mechanical Alloying. J. Alloys Compd..

[28] Yang Y., Licavoli J.J., Sanders P.G. (2020). Improved Strengthening in Supersaturated Al-Sc-Zr Alloy via Melt-Spinning and Extrusion. J. Alloys Compd..

[29] Taha M.A., El-Mahallawy N.A., Abdel-Ghaffar M.A., Klaar J. (1991). Morphological Variations in Melt-Spun Al Cu Alloys. Mater. Sci. Eng. A.

[30] Crisan A.D., Leca A., Bartha C., Dan I., Crisan O. (2021). Magnetism and ε-τ Phase Transformation in MnAl-Based Nanocomposite Magnets. Nanomaterials.

[31] Crisan A.D., Leca A., Dan I., Crisan O. (2021). Thermal Stability, Blocking Regime and Superparamagnetic Behavior in Mn-Al-C Melt Spun Ribbons. Nanomaterials.

[32] Çağlar S. (2025). Impact of B_4_C Reinforcement on the Microstructure, Wear, Hardness, Corrosion Behavior, and Radiation Shielding Properties of Al-40Sm_2_O_3_ Hybrid Composites. Nucl. Eng. Technol..

[33] Liu Y., Xie J., Peng H., Liu C., Ma D., Leng Y. (2025). Effects of Boron Addition on Microstructure and Mechanical Properties of B_4_C/Al Composites Fabricated by Pressureless Infiltration. Metals.

[34] Wu S., Wu S., Xiao G., Xue L., Zhai M., Zhu W. (2015). Solid Reaction between Al and B_4_C. Can. Metall. Q..

[35] Scherrer P. (1918). Nachrichten von der Gesellschaft der Wissenschaften zu Göttingen. Math.-Phys. Kl..

[36] Guo R.-F., Chen S.-M., Shen P. (2023). Influence of Si, Ti, and Cu as Alloying Elements on the Wettability and Reactivity of an Al/B_4_C System. J. Mater. Res. Technol..

[37] Çağlar S. (2025). Corrosion Behavior, Microstructure, and Mechanical Properties of Al-10Sm_2_O_3_-B Neutron Shielding Composites. Nucl. Mater. Energy.

[38] Caglar S., Kilicaslan M.F., Atasoy A., Tiryaki H., Erkovan M., Hong S.-J. (2021). Effect of Heat Treatment on Magnetic Properties of Nanocomposite Nd-Lean Nd_7_Fe_73_B_20_ Ribbons. J. Mater. Sci. Mater. Electron..

[39] Bayrak Y., Özbay Kısasöz B., Tarakçı G., Kısasöz A. (2024). Characterization of Al/B_4_C–Y_2_O_3_ Hybrid Composites Produced by Vacuum Hot Pressing Combined with Al_2_O_3_–Y_2_O_3_ Interaction. Ceram. Int..

[40] Çanakçı A., Karabacak A.H., Çelebi M., Özkaya S., Arpacı K.A. (2024). A Study on the Optimization of Nano-B_4_C Content for the Best Wear and Corrosion Properties of the Al-Based Hybrid Nanocomposites. Arab. J. Sci. Eng..

[41] Hssissou R., Benzidia B., Hajjaji N., Elharfi A. (2019). Elaboration, Electrochemical Investigation and Morphological Study of the Coating Behavior of a New Polymeric Polyepoxide Architecture: Crosslinked and Hybrid Decaglycidyl of Phosphorus Pentamethylene Dianiline on E24 Carbon Steel in 3.5% NaCl. Port. Electrochim. Acta.

[42] Ozturk K., Gecu R., Karaaslan A. (2021). Microstructure, Wear and Corrosion Characteristics of Multiple-Reinforced (SiC–B_4_C–Al_2_O_3_) Al Matrix Composites Produced by Liquid Metal Infiltration. Ceram. Int..

[43] Aydın F. (2023). A Review of Recent Developments in the Corrosion Performance of Aluminium Matrix Composites. J. Alloys Compd..

